# S-ketamine facilitates motor function recovery after brachial plexus root avulsion and reimplantation in mice

**DOI:** 10.3389/fphar.2025.1630158

**Published:** 2025-07-23

**Authors:** Ronghua Huang, Bingbiao Lin, Lingtai Yu, Qichen Luo, Hongyan Tian, Chenrui Li, Naili Wei, Shaohui Zhuang, Jian Chen, Yalan Li

**Affiliations:** ^1^ Department of Neurosurgery, The First Affiliated Hospital of Shantou University Medical College, Shantou, China; ^2^ Department of Anesthesiology, The First Affiliated Hospital of Jinan University, Guangzhou, China; ^3^ Department of Radiotherapy, Cancer Hospital of Shantou University Medical College, Shantou, China; ^4^ Laboratory of Biomaterials and Translational Medicine, Center for Nanomedicine, Department of Urology, The Third Affiliated Hospital, Sun Yat-sen University, Guangzhou, China; ^5^ Key Laboratory of CNS Regeneration (Ministry of Education), Guangdong-Hongkong-Macau CNS Regeneration Institute of Jinan University, Guangzhou, China; ^6^ Department of Anesthesiology, The First Affiliated Hospital of Shantou University Medical College, Shantou, China; ^7^ Department of Anesthesiology, Guangzhou Concord Cancer Center, Guangzhou, China

**Keywords:** S-ketamine, brachial plexus root avulsion, motor function, recovery, BDNF

## Abstract

**Background:**

Brachial plexus root avulsion (BPRA) often occurs in high-speed traffic accidents or shoulder dystocia, resulting in motor dysfunction. S-ketamine, a clinical anesthetic and antidepressant drug, is an NMDA receptor antagonist that may be effective against glutamate excitotoxicity after nerve injury. Therefore, we aimed to elucidate the potential effectiveness of S-ketamine on motor function recovery after BPRA in mice.

**Methods:**

A mouse model of BPRA and reimplantation was established, and mice were randomly assigned to either the S-ketamine group or the control group, receiving a low, subanesthetic dose of S-ketamine or normal saline, respectively. The restoration of the motor neural circuit—from spinal cord and myocutaneous nerve to biceps muscle—was evaluated. Fluoro-Gold retrograde tracing was utilized to assess the connectivity between the central and peripheral nerve systems. Behavioral tests such as CatWalk, grooming test, and grip strength were applied to assess motor function recovery. The underlying mechanism was analyzed by Western blot, and the rescue experiment was assessed via motor function behavioral tests.

**Results:**

S-ketamine increased motor neuron survival, enhanced central and peripheral nervous connectivity, promoted axon regeneration and remyelination, improved the neuromuscular junction integrity, and prevented muscle atrophy. As a result, motor function recovery was significantly improved, which was attributed to increased BDNF production via ERK-CREB phosphorylation. The BDNF receptor antagonist, ANA12, counteracted the functional recovery induced by S-ketamine.

**Conclusion:**

S-ketamine increases the BDNF concentration by ERK/CREB phosphorylation, thereby promoting motor neural circuit repair and facilitating motor function recovery.

## Graphical Abstract

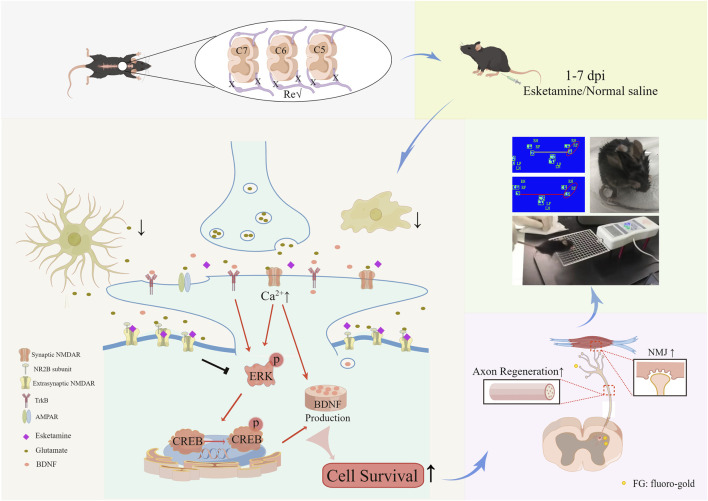

## Introduction

Brachial plexus root avulsion (BPRA) is a severe form of brachial plexus injury that typically results from traumatic events such as high-velocity accidents or shoulder dystocia, with motor traffic accidents being the leading cause, particularly in developing countries (Eggers et al., 2020; Faglioni et al., 2013; Kaijomaa et al., 2022). BPRA involves the complete detachment of one or more nerve roots from the spinal cord, leading to significant sensory and motor deficits in the affected upper limb. Nerve tissue possesses limited ability to regenerate following injury, making the functional recovery of damaged nerves after surgery challenging to accomplish (Yao et al., 2021; Yao et al., 2024). Moreover, abnormal axonal regeneration and organization cause neuroma, leading to hyperpathia or intolerable pain (Yu A.-X. et al., 2023). Currently, BPRA remains a complex challenge in reconstructive surgery as no surgical repair technique guarantees the full recovery of motor function in the hands (Fang et al., 2016; Lundborg and Rosén, 2007). Surgery combined with pharmacological treatment to improve nerve regeneration may be a novel approach to overcome the limitations and side effects and, most importantly, to achieve better outcomes (Hui et al., 2022).

Glutamate excitotoxicity is a pathological process where excessive glutamate, the primary excitatory neurotransmitter in the central nervous system (CNS), overstimulates the neurons and leads to cell injury or death (Lai et al., 2014; Parsons and Raymond, 2014). This phenomenon often occurs after neuronal injury, and it is a major contributor to secondary damage in the CNS. Excessive release of glutamate to the extracellular space overactivates the glutamate receptors, such as the N-methyl-D-aspartate receptors (NMDARs), which are permeable to calcium ions (Ca^2+^), and their overstimulation results in a massive influx of Ca^2+^ into the neurons (Parsons and Raymond, 2014). As a result, elevated intracellular Ca^2+^ triggers several harmful biochemical pathways and eventually causes neuron death and further glutamate release, creating a detrimental cycle (Hardingham et al., 2002).

The Ca^2+^ influx via the synaptic NMDAR facilitates the transmission of neuro-electrochemical signals, activating Ca^2+^-dependent calmodulin kinases and the Ras-ERK1/2 pathway. This promotes the activation of the downstream nuclear transcription factor, cAMP response element-binding protein (CREB). Activated CREB further enables the transcription and translation of target proteins such as BDNF, completing the signal feedback loop (Hardingham and Bading, 2010; Hardingham et al., 1999; Wu et al., 2001; Wang et al., 2016). However, extrasynaptic NMDAR activation may cause the opposite effect, resulting in neuron death (Parsons and Raymond, 2014; Hardingham et al., 2002; Ivanov et al., 2006).

S-ketamine, a glutamate NMDAR antagonist, is the focus of recent attention due to its novel, fast-acting pharmacotherapies for depression (McIntyre et al., 2021; Reif et al., 2023; Daly et al., 2019). The underlying mechanism is that S-ketamine can serve as an NMDAR modulator and increase the BDNF concentration in the prefrontal cortex and hippocampus (Aleksandrova and Phillips, 2021; Zanos and Gould, 2018; Kavalali and Monteggia, 2012). Therefore, we hypothesized that S-ketamine may treat the glutamate excitotoxicity and promote recovery after BPRA using a mouse model (Lai et al., 2014).

## Materials and methods

### Animals

Animal experiments were performed according to the guidelines that have been approved by the Ethics Committees of Jinan University, Guangdong, China (approval number: IACUC-20220121-02). Animals were housed at 23 °C ± 1 °C under a 12-h dark/light cycle and provided free access to water and food.

### BPRA model

Eight-week-old male C57BL6/J mice were used to establish the BPRA and replantation models, as previously described (Yu L. et al., 2023). In brief, the longest spine, T2, was used as an anatomical marker to locate the C5–7 vertebrae. Following unilateral hemilaminectomy, the right C5–7 dorsal and ventral roots were surgically removed. To imitate clinical surgery treatment, the C6 root was reimplanted into its original location. Mice were kept on a warm pad to maintain their body temperature until they woke up.

### Treatment

Model mice were randomly divided into the S-ketamine group or the control group, receiving intraperitoneal injection of S-ketamine or an equal volume of saline, respectively, for seven consecutive days after BPRA. In the second set of designs, mice were randomly divided into the following four groups and received the corresponding treatment: control (saline), S-ketamine (10 mg/kg), ANA12 (0.5 mg/kg), and EA (0.5 mg/kg ANA12 + 10 mg/kg S-ketamine).

### Behavioral tests

#### Grooming tests

Water was sprayed on the faces of the mice. A video camera was used to record the movements of the forelimbs for five consecutive 5 minutes. The grooming score of the right forelimb was evaluated according to the following criteria: 0, no movement; 1, shaking but not reaching the chin; 2, reaching the chin but not the cheek; 3, reaching the cheek but not beyond the right eye; 4, reaching the right eye but not beyond the right ear; and 5, reaching the right ear.

#### Grip strength tests

The strength of the right forelimb was assessed using a grip strength meter (NO47200; Ugo Basile; Italy). Mice underwent five testing sessions, with a 10-min interval between each.

#### CatWalk

Mice were placed in an enclosed glass walkway, and a camera was positioned 40 cm behind the walkway to record the footprints. Runs from one side to the other side within 10 s without climbing were defined as completed runs. Data from the completed runs, such as the velocity, print area, stride length, and swing speed, were analyzed using Noldus Software (Netherlands).

### Histology and immunofluorescence

Spine and biceps muscles were dissected after transcardial perfusion of PBS and 4% paraformaldehyde (4% PFA) of the mice. Samples were post-fixed in 4% PFA overnight and then dehydrated in 10%, 20%, and 30% sucrose, after which they were cut by a cryostat microtome (Thermo Fisher Scientific) into 30 μm/slice.

After being blocked with 3% bovine serum albumin and 10% donkey serum in 0.3% PBST for 2 h, the slices were incubated overnight with the primary antibody at 4 °C. After being washed with PBS three times the next day, slices were incubated with secondary antibody for 2 h at room temperature. The antibodies used in this study included anti-ChAT (Sigma, AB144P), anti-Iba1 (Wako, 019-19741), anti-GFAP (Abcam, 4674), anti-NF200 (Sigma, N0142), α-Bungarotoxin, Alexa Fluor 594 conjugate (Invitrogen, B13422), Alexa Fluor 488 Donkey Anti-Chicken (Jackson, 112117), Alexa Fluor 647 Donkey anti-Rabbit (Invitrogen, A-31573), Alexa Fluor 647 Donkey anti-mouse (Invitrogen, A-31571), and Alexa Fluor 647 Donkey anti-goat (Invitrogen A-11058).

### Western blot

Proteins were extracted using RIPA lysis buffer (Beyotime, P0013B) with protease and phosphatase inhibitor cocktail (Beyotime, P1045) on ice, homogenized via ultrasonication, and centrifuged at 12,000 × *g* for 15 min at 4 °C. The supernatant was quantified using a BCA assay kit (Beyotime, P0012). Samples were then subjected to 8%–12% SDS-PAGE gel (Beyotime, P0012A) and transferred to polyvinylidene fluoride (PVDF) membranes (Merck Millipore). Membranes were blocked with 5% BSA in 0.1% TBST (TBS with 0.1% Tween 20) for 2 h at room temperature and then incubated with the primary antibodies overnight at 4 °C. After washes in 0.1% TBST, the membranes were incubated with secondary antibodies for 2 h at room temperature. The blots were visualized using a ChemiDoc Touch Imaging System (Bio-Rad) and quantified using ImageJ.exe. The antibodies used included CREB (PTM5595), p-CREB (Santa Cruz sc-81486), ERK (CST 4695), p-ERK (CST 4370), BDNF (Abcam 108319), HRP goat anti-rabbit (1:5000, Abcam, ab6721), and HRP goat anti-mouse (1:5,000, Abcam, ab6789).

### Electron microscopy

Myocutaneous nerves at 8 weeks after BPRA were prepared for electron microscopy (EM) studies. Briefly, animals were perfused with 2.5% glutaraldehyde (Sigma) plus 2% paraformaldehyde, and then 2-mm distal myocutaneous nerves were collected for post-fixation at 4°C overnight. Samples were cut into semi-thin sections and ultrathin sections. Semi-thin (500 nm) transverse sections were captured under a ×63 oil objective. For ultrastructural analysis, 50-nm ultrathin sections were prepared and captured in ×3,000 and ×12,000 objectives using a Philips 400 transmission electron microscope. The number of different-sized axons and G-ratio (the inner/outer diameter of the myelin sheath) were measured using ImageJ.

### Retrograde tracing

On the 53rd day post injury (dpi53), bilateral myocutaneous nerves were injected with 1 μL 6% Fluoro-Gold using a Hamilton microsyringe. C5–7 spinal segments were dissected after 3 days and cut into 30-μm sections. Fluoro-Gold-labeled neurons were captured using a Zeiss microscope.

### Statistical analysis

The data are presented as the mean ± SEM. After confirming the homogeneity of variance with Levene’s test, intergroup comparisons were performed using two-tailed unpaired Student’s t-tests in GraphPad Prism (version 8.0.1). *P* < 0.05 was regarded as significant.

## Results

1. S-ketamine ameliorates the reactive astrocyte and microglial activation on the injured side of the spinal cord.

**FIGURE 1:**
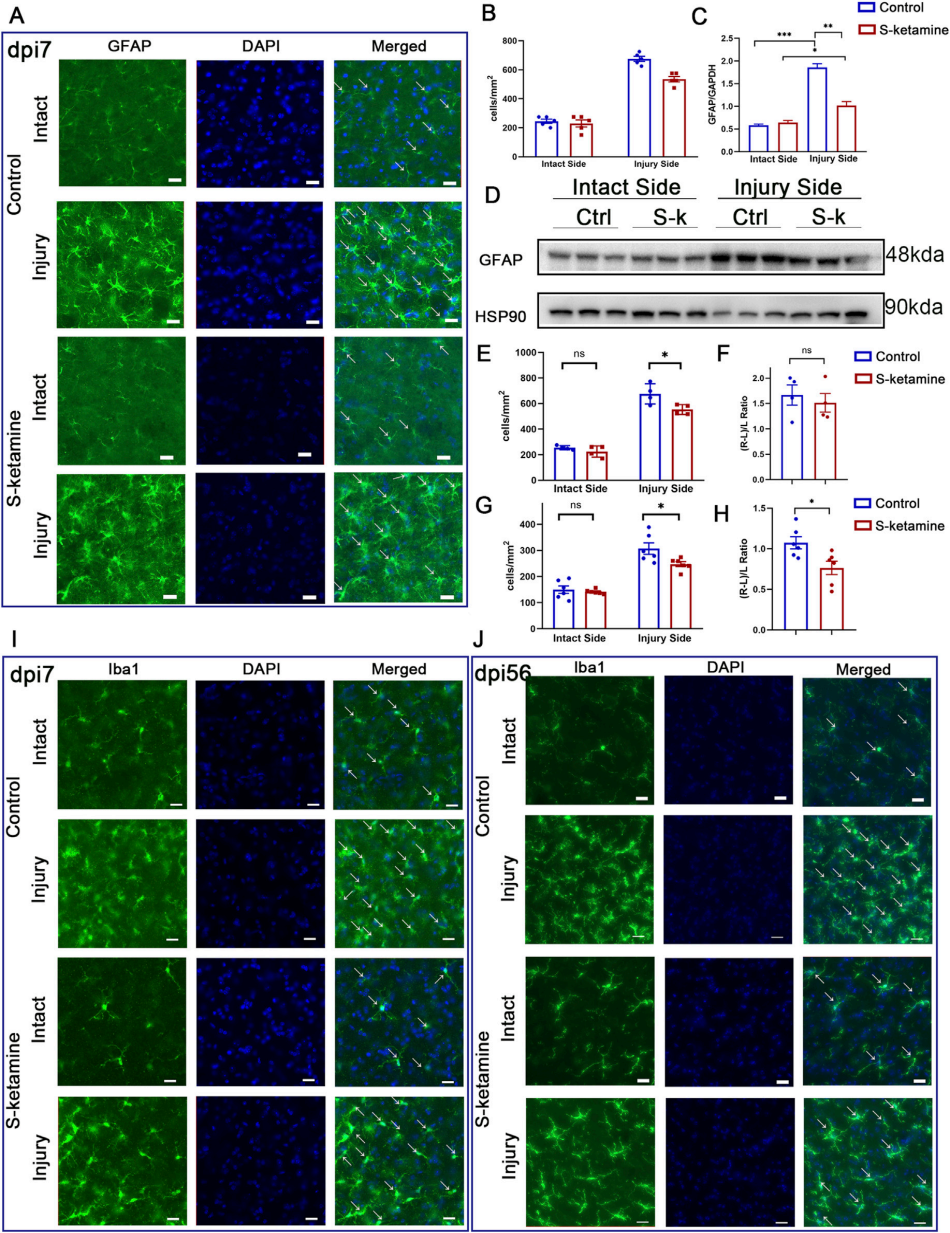
S-ketamine ameliorates reactive astrocyte and microglial activation on the injured side of the spinal cord. **(A)** Immunofluorescence of the GFAP-labeled astrocytes of the spinal cord in the control and S-ketamine groups. **(B)** Quantification of cell densities of GFAP-labeled astrocytes. **(C, D)** GFAP protein expression via Western blotting and quantification of the blots. **(E, F)** Cell density and proliferative rate of microglia in the spinal cords on dpi7. **(G, H)** Cell density and proliferative rate of microglia in the spinal cords on dpi56. **(I, J)** Immunofluorescence of microglia on dpi7 and dpi56, respectively. The statistics are shown as the mean ± SEM. ***p < 0.001, **p < 0.01, and *p < 0.05. ns, non-significant; dpi, days post-injury; Ctrl, control; S-k, S-ketamine.

In response to central nervous system injury, astrocytes become activated and proliferative, which is known as reactive astrogliosis. Immunofluorescence and Western blotting were utilized to assess the extent of damage after BPRA. The results showed that astrocytes on the injured side of the spinal cord were significantly activated, and the number was dramatically increased (Figures 1A, B), along with the elevated GFAP protein expression compared to that on the intact side (Figures 1C, D). However, treatment with S-ketamine effectively reduced reactive astrogliosis, as evidenced by a decrease in both the astrocyte number and GFAP protein expression compared to that in the control group.

As the resident immune cells in the CNS, microglia exhibited amoeboid morphological changes after injury. Moreover, overactivated microglia can lead to an increase in neurotoxic astrocytes, further exacerbating neuronal death. In the acute phase (dpi 7), microglia density on the intact side of the two groups was 254.6 ± 7.9 cells/mm^2^ and 225.0 ± 21.8 cells/mm^2^, respectively, without significant difference (Figures 1E, I). On the contrary, both groups exhibited significant microglial activation on the injured side, with densities increasing by 1.7 ± 0.2 times and 1.5 ± 0.2 times compared to that on the intact side (Figure 1F). Nevertheless, S-ketamine treatment led to a significant reduction in microglial cell density on the injured side compared to that in the control group (553.6 ± 19.4 cells/mm^2^ vs. 675.4 ± 39.7 cells/mm^2^, *p* = 0.03) (Figures 1E, I). These findings indicate that S-ketamine could alleviate the overactivation of microglia after BPRA.

In the chronic recovery phase (dpi 56), both groups of mice still exhibited a certain level of microglial activation on the injured side compared to the intact side, but the activation gradually returned to baseline levels. The S-ketamine-treated group showed better recovery in microglial activation than the control group (microglial proliferation rate: 0.764 ± 0.083 vs. 1.074 ± 0.074, *p* = 0.02) (Figure 1H). Additionally, there was a significant difference in microglial density on the injured side between the two groups, with the treatment group showing a marked reduction in spinal cord microglia compared to the control group (247.6 ± 9.3 cells/mm^2^ vs. 307.2 ± 22.0 cells/mm^2^, *p* = 0.03) (Figures 1G, J). These findings indicate that S-ketamine suppressed the overactivation of microglia.

2. S-ketamine increases the motor neuron survival.

**FIGURE 2:**
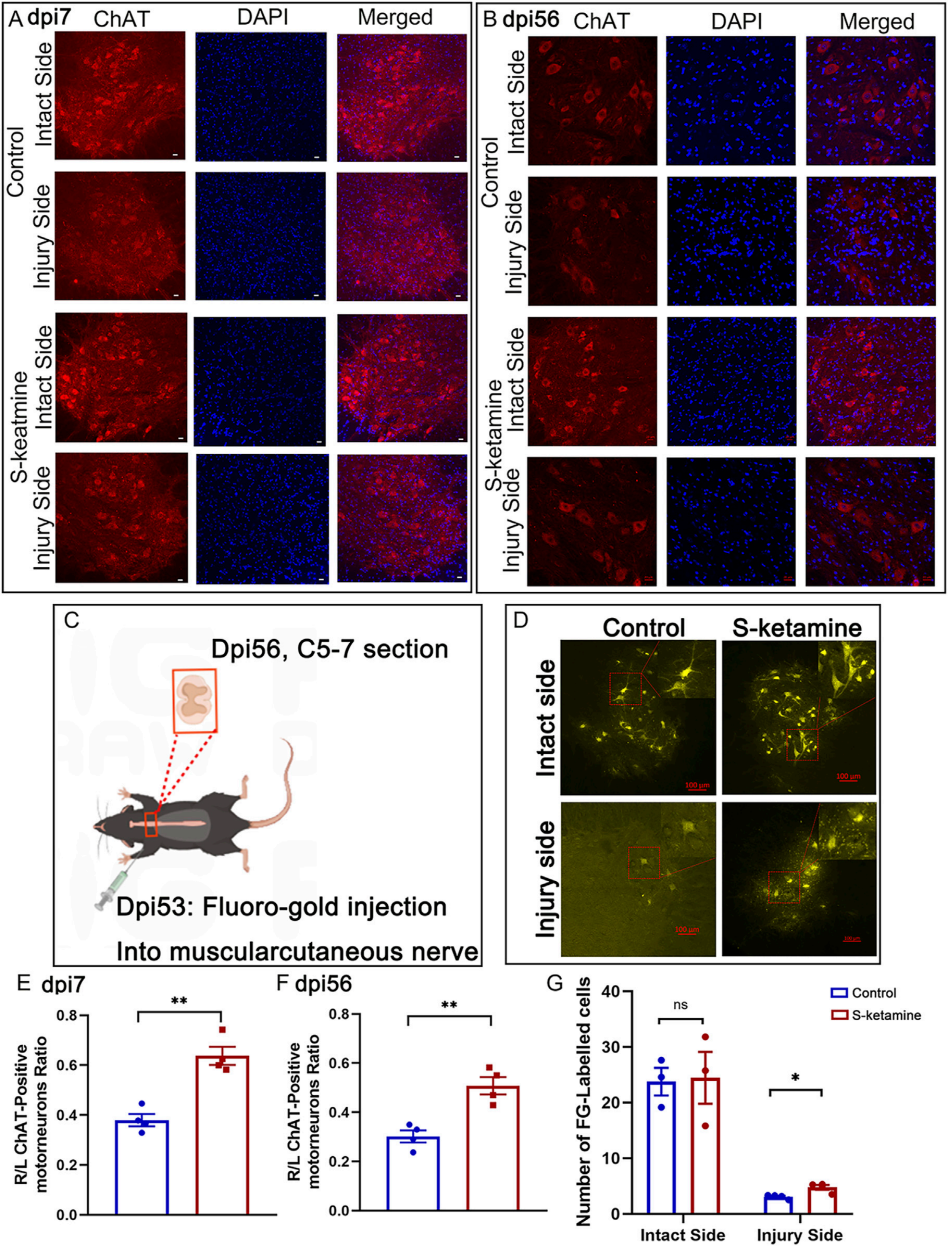
S-ketamine elevates motor neuron survival and strengthens the connectivity between the central and peripheral nerves. **(A, B)** Immunofluorescence of motor neuron on dpi7 and dpi56, respectively. **(C)** Protocol of Fluoro-Gold retrograded experiment. **(D)** Fluoro-Gold-labeled neurons in the spinal cords. **(E, F)** Survival rate of motor neurons compared to the intact sides of the two groups on dpi7 and dpi56, respectively. **(G)** Quantification of Fluoro-Gold-labeled neurons. The statistics are shown as the mean ± SEM. **p < 0.01 and *p < 0.05. dpi, days post-injury.

Nerve root injury may cause motor neuron death of the corresponding segmented spinal cord. During the acute phase, the motor neurons became swollen, and most underwent death, though S-ketamine treatment effectively reduced neuron death (0.637 ± 0.036 vs. 0.380 ± 0.025, *p* < 0.01) (Figures 2A, E). Only one-third of the motor neurons in the control group survived 8 weeks after the model surgery, whereas nearly half survived in the S-ketamine group (0.508 ± 0.035 vs. 0.302 ± 0.025, *p* < 0.01) (Figures 2B, F). S-ketamine significantly ameliorates neuron death in the acute phase, thereby increasing the survival rate of motor neurons in the long-term period.

3. S-ketamine promotes axon regeneration and remyelination.

Surviving neurons possess a certain capacity for axon regeneration; therefore, Fluoro-Gold retrograde tracing was utilized. Three days after bilateral myocutaneous nerve injection of Fluoro-Gold in dpi53 (Figure 2C), we found Fluoro-Gold-labeled motor neurons in the C5–7 spinal cord, especially on the intact side, without significant difference between the two groups (S-ketamine group 24.5 ± 4.7 cells/section vs. control group 23.8 ± 2.5 cells/section, *p* = 0.904) (Figures 2D, G). Meanwhile, on the injured side, few Fluoro-Gold-labeled motor neurons were found, but significantly more were found in the S-ketamine-treated group than in the control group (4.8 ± 0.4 cells/section vs. 3.1 ± 0.2 cells/section, *p* = 0.01) (Figures 2D, G).

**FIGURE 3:**
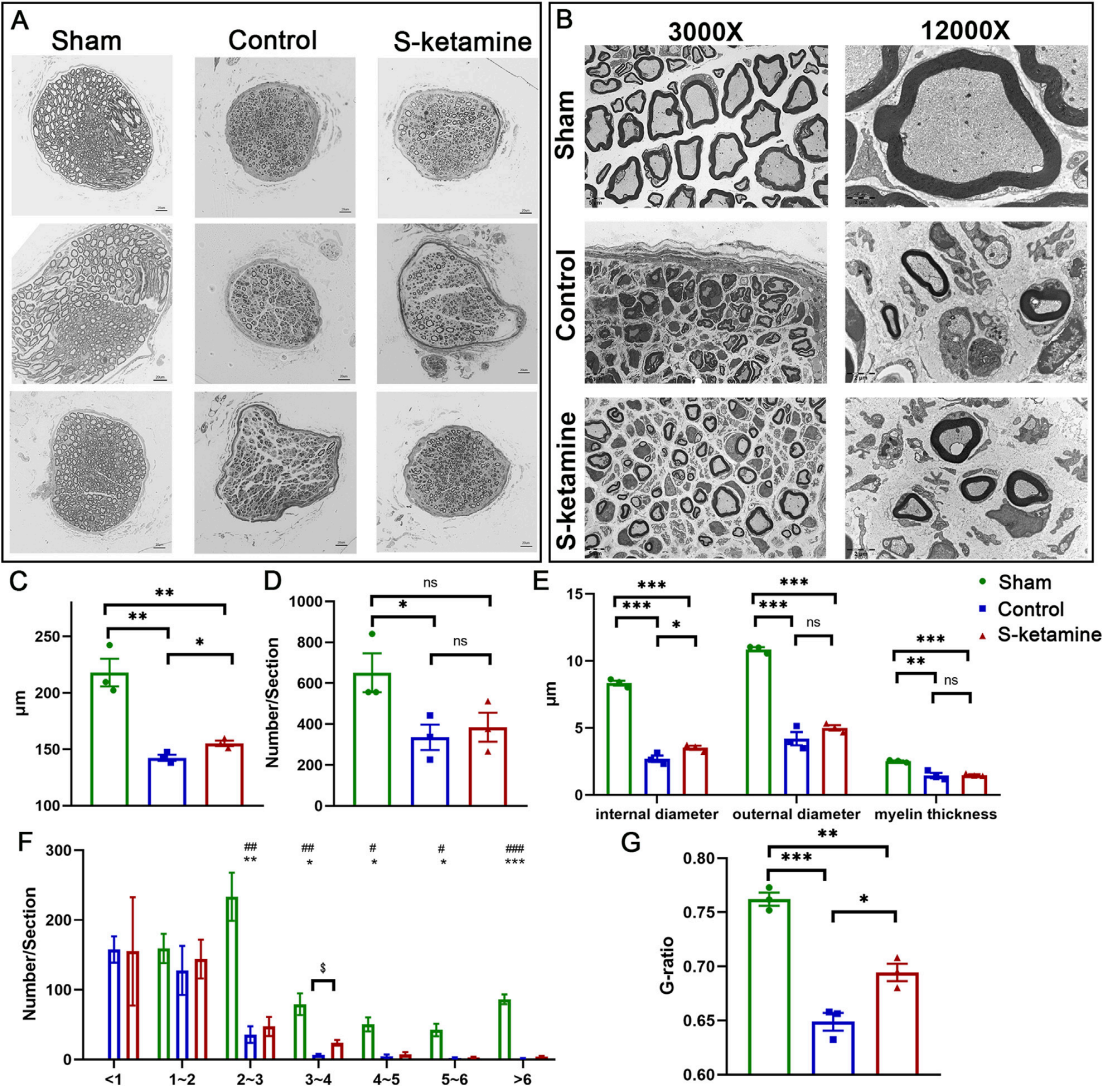
S-ketamine promotes axon regeneration and remyelination. **(A)** Morphology of myocutaneous nerve and internal axons. **(B)** Neural diameter. **(C)** Axon number per section. **(D)** Internal and external diameter and thickness of the myelin sheath. **(E)** Morphology of myelin sheath via electron microscopy. **(F)** Number of axons in different ranges of the diameter. **(G)** G-ratio of the myelin sheath. *** and ^###^, *p* < 0.001; ** and ^##^, *p* < 0.01; and * and ^#^, *p* < 0.05. ns, non-significant.

Despite axon regeneration, the number of inner axons and remyelination are crucial for nerve innervation. Semi-thin sections showed that the myocutaneous nerve became dramatically atrophied after BPRA, which was reversed by S-ketamine to some extent (Figure 3A). On one hand, the neural diameter was 218.0 ± 12.3 μm in the sham group but 142.4 ± 2.7 μm in the control group; fortunately, the diameter in the S-ketamine group was slighter larger (155.2 ± 2.5 μm, *p* < 0.05) (Figure 3B). On the other hand, the total axon number inside the myocutaneous nerve was sharply reduced after BPRA although there is a trend that more axons were found in the S-ketamine group than in the control group (385 ± 71/section vs. 335 ± 62/section) (Figure 3C). In addition, we counted the number of axons with different diameters (Figure 3D). The diameter of all the axons in the sham group was larger than 1 μm, and most of the axon diameters were 2–3 μm. On the contrary, most of the axon diameters in the control and S-ketamine groups were smaller than 1 μm. However, in diameter ranges greater than 1 μm, the number of axons was greater in the S-ketamine group than in the control group, especially with significance in the 3–4 μm range.

Ultrathin sections revealed the remyelination of the regenerated axon (Figure 3E). Compared to that of the sham group, both the inner and outer diameters of the nerve fiber and myelin thickness were significantly thinner after BPRA. Meanwhile, the inner diameter, which revealed the axon diameter, was remarkably larger in the S-ketamine group than in the control group (3.523 ± 0.154 μm vs. 2.701 ± 0.244 μm, *p* < 0.05) (Figure 3F). Referring to the efficiency of nerve conduction, the G-ratio of the myocutaneous nerve in the sham group was 0.762 ± 0.006, which is similar to other researchers’ reports (Duval et al., 2017; Benninger et al., 2006). A significant reduction in the G-ratio was found after BPRA (control: 0.649 ± 0.008, *p* < 0.001; S-ketamine: 0.694 ± 0.008, *p* < 0.01), though the G-ratio of the nerve in the S-ketamine group was closer to that of the sham group (*p* < 0.05) (Figure 3G).

4. S-ketamine reversed biceps atrophy and improved neuromuscular junction (NMJ) morphology.

**FIGURE 4:**
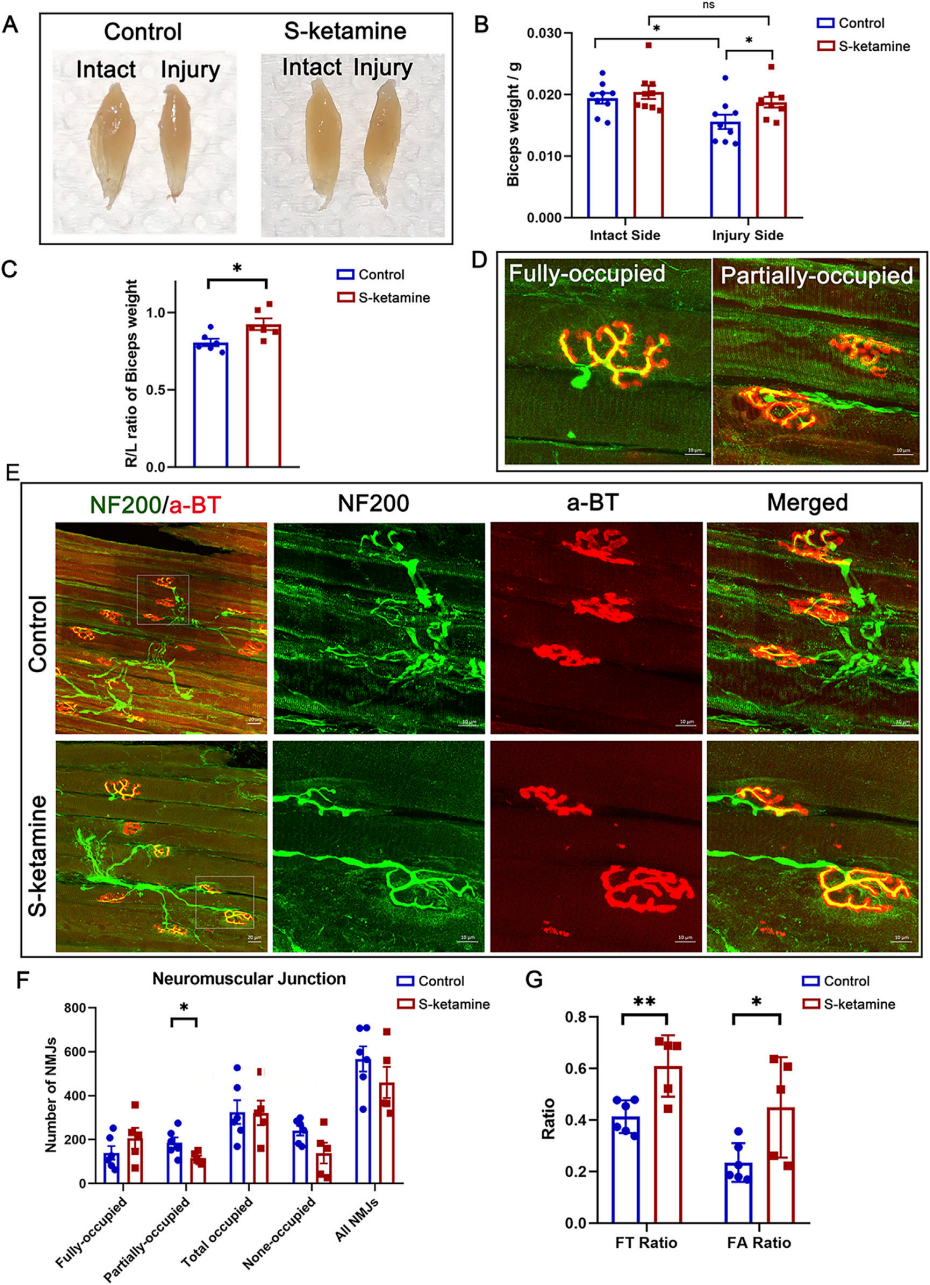
S-ketamine reverses muscle atrophy and preserves NMJ integrity. **(A)** Representative photographs of biceps muscles. **(B)** Weight of the biceps. **(C)** Ratio of the ipsilateral muscle weight to that of the contralateral sides. **(D)** Definition of the fully and partially occupied NMJs. **(E)** Immunofluorescence of NF200 and α-BT-labeled NMJs inside the biceps on the injured sides. **(F)** Number of different types of NMJs. **(G)** FT ratio (fully occupied NMJs to total-occupied NMJs) and FA ratio (fully occupied NMJs to all NMJs) of the two groups. NMJ, neuromuscular junction. *p < 0.05.

Without nerve innervation after BPRA, the biceps on the injured side became atrophied, as the wet weight was much lower than that of the intact side. S-ketamine treatment elevated the wet weight of the biceps and reduced the atrophy degree (R/L ratio of biceps weight: control 0.81 ± 0.00 vs. S-ketamine 0.93 ± 0.00, *p* = 0.025) (Figures 4A–C).

Furthermore, NMJs were labeled using NF200 (nerve marker) and α-BT (motor end-plate (MEP) marker). According to the innervation degree of NF200, all MEPs were classified as fully occupied, partially occupied, and non-occupied MEPs (Figure 4D). Figure 4E revealed more integrated NMJs in the S-ketamine group, and different types of MEPs were quantified (Figure 4F). In addition, total-occupied MEPs were defined as both fully and partially occupied MEPs, and the total number included all types of MEPs. There is a trend that more fully-occupied MEPs were found in the S-ketamine group. Moreover, the FT ratio (fully-occupied/total-occupied MEPs) (0.609 ± 0.053 vs. 0.413 ± 0.026, *p* < 0.01) and the FA ratio (fully-occupied/all MEPs) (0.449 ± 0.087 vs. 0.235 ± 0.031, *p* = 0.03) were higher in the S-ketamine group than in the control group (Figure 4G).

5. S-ketamine facilitates the function of the biceps after BPRA in mice.

**FIGURE 5:**
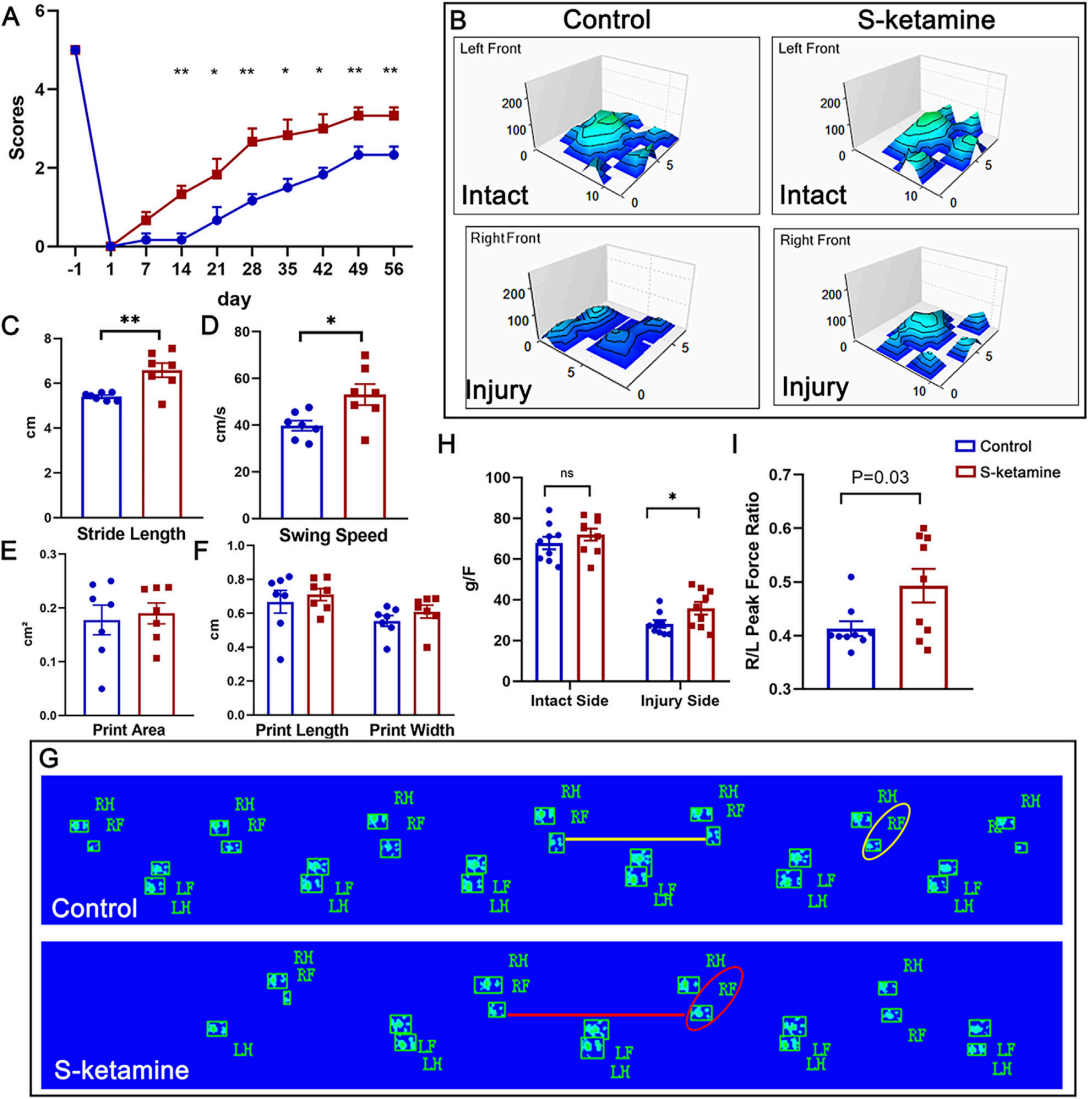
S-ketamine promotes motor function recovery after BPRA and reimplantation in mice. **(A)** Weekly grooming test score. **(B)** 3D representation of footprint intensity charts. **(C–F)** Quantification of the stride length, swing speed, print area, print length, and print width of the injured sides between the two groups. **(G)** Footprint of the mice in the control and S-ketamine groups on dpi56. **(H)** Peak force of the forelimbs. **(I)** Ratio of the peak force of the injured forelimb to that of the intact side. **p < 0.01 and *p < 0.05.

The grooming test was used to assess the functional recovery of the elbow flexion (Figure 5A). Previous studies have shown that the grooming scores of mice can reach more than two points after BPRA. In our study, both groups of mice exhibited increasing scores after BPRA and reimplantation, reaching a plateau at 7 weeks post-injury. The grooming score in the control group was 2.167 ± 0.307 points at dpi49. In contrast, the S-ketamine treatment group showed a significantly higher grooming score of 3.333 ± 0.211 points (*p* = 0.011). Furthermore, starting from week 3 post-injury, the treatment group consistently demonstrated significantly higher grooming scores than the control group.

In addition, CatWalk recording was performed to analyze spontaneous movement (Figure 5G). The palm and finger prints of the left intact forepaws were clearly identified in both groups, as shown in the three-dimensional (3D) footprint intensity charts (Figure 5B). The prints of the forepaws in the control group were not as easily recognized as those in the S-ketamine group. In addition, the stride length was significantly shorter and the swing speed was markedly slower in the control group than those in the S-ketamine group, whereas the print length, print width, and print area showed no significant differences (Figures 5C–F).

Finally, we utilized the grip strength test to validate the comprehensive ability of the injured forelimb on days 7, 14, 28, and 56 post-injury. A sharp reduction in strength was found on the injured sides compared to the intact sides. Fortunately, mice in the S-ketamine group showed higher strength than those in the control group on dpi56 (35.767 ± 3.087 g vs. 28.111 ± 1.935 g, *p* = 0.049) (Figure 5H). Consistently, the right-side peak force over the left-side ratio was much greater in the S-ketamine group (0.493 ± 0.031 vs. 0.413 ± 0.014, *p* = 0.03) (Figure 5I).

6. S-ketamine increases the BDNF concentration by reversing ERK/CREB phosphorylation.

**FIGURE 6:**
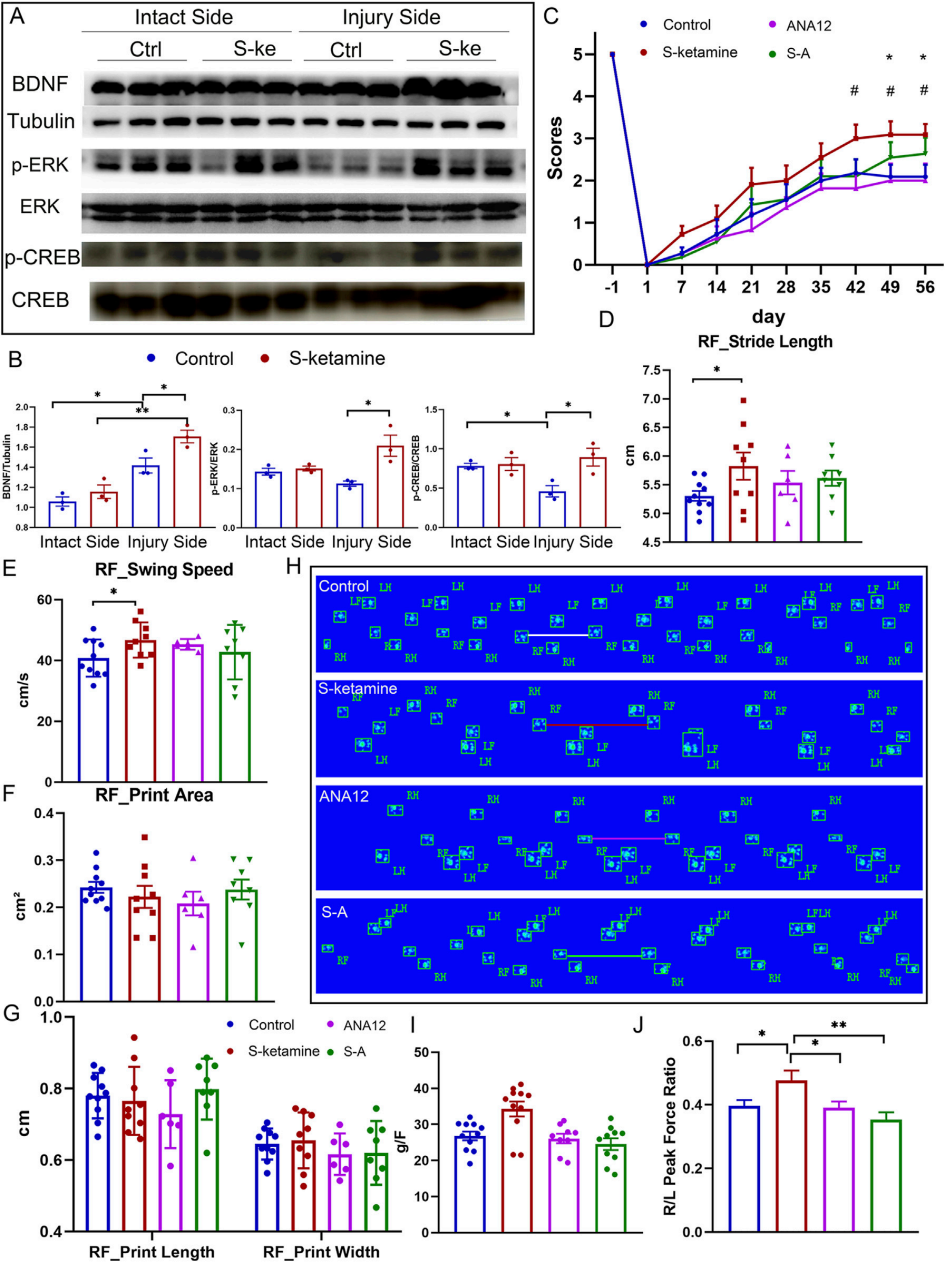
S-ketamine elevates the BDNF concentration by reversing ERK/CREB phosphorylation. **(A, B)** Western blot of BDNF, p-ERK, ERK, p-CREB, CREB, tubulin, and quantification of the blots. **(C)** Weekly grooming test scores of the mice in each group. **(D–G)** Quantification of the stride length, swing speed, print area, print length, and print width of the injured side in each group. **(H)** Footprints of the mice in each group on dpi56. **(I)** Peak force of the forelimbs in each group. **(J)** Ratio of the peak force of the injured forelimb to that of the intact side in each group. **, *p* < 0.01 and * and ^#^, *p* < 0.05. Ctrl, control; S-k, S-ketamine.

The reactive increase in BDNF is one of the neuronal self-rescue mechanisms after injury. As expected, the BDNF concentration was much higher on the injured sides of the spine than on the intact sides, and S-ketamine further elevated the BDNF concentration of the injured side (Figure 6A).

Extrasynaptic NMDAR activation may silence ERK/CREB signaling. We found that ERK/CREB phosphorylation was inhibited in the injured spinal cord. However, S-ketamine reversed ERK/CREB phosphorylation, which significantly supported neuron survival (Figures 6A, B).

To validate the role of BDNF in S-ketamine’s effect on neuronal injury, we used ANA12, the antagonist of the BDNF receptor TrkB. As shown in Figures 6C–J, ANA12 counteracted the motor function recovery using S-ketamine alone. The grooming score and grip strength in the S–A group were reduced compared to those in the S-ketamine group. Moreover, the stride length was significantly shorter and the swing speed was markedly slower in the S–A group.

## Discussion

In this experiment, we used a mouse model to mitigate brachial plexus root avulsion and reimplantation after injury, and mice successfully displayed abnormalities in the affected limbs. Motor function showed little recovery after reimplantation in the control group. With S-ketamine administration, behavioral deficits observed in the grooming test, grip strength, and CatWalk analysis were improved compared to those in the control group. We further validated that this effect was due to the elevation of the BDNF concentration mediated by ERK/CREB phosphorylation. To our knowledge, the effect of S-ketamine on BPRA injury is an innovative finding.

Nerve regeneration after trauma is a complex biological process influenced by various biological and environmental factors, including nerve cell survival, the rate of axonal regeneration, the integrity of the NMJ, the degree of de-innervation and muscle atrophy, the patient’s age, and adherence to rehabilitation (Lundborg and Rosén, 2007). The motor function recovery relies on the restoration of the motor neural circuit’s integrity, for which neuron survival serves as the cornerstone. Interventions capable of boosting neuro-restorative processes and adaptive neuroplasticity play a pivotal role in long-term disability prevention and functional recovery enhancement (Pisarchik et al., 2024). Previous studies revealed that the motor neuron survival range was approximately 30%–35% after BPRA and reimplantation (Guo et al., 2019; Chen et al., 2019; Tang et al., 2019), similar to our result in the control group (30.2%). However, S-ketamine significantly elevated neuron survival to 50.8%, probably due to the relief of astrocyte and microglia overactivation. Moreover, axon regeneration and remyelination were promoted with S-ketamine administration as the myocutaneous nerve was appeared thicker and contained more axons than in the control group, especially in the 2–3 μm axon range, and the G-ratio of the myelin was closer to the normal value. In addition, Fluoro-Gold retrograde tracing revealed that more Fluoro-Gold-labeled motor neurons were found in the S-ketamine group, indicating the tighter connectivity between the central and peripheral nervous systems. Finally, with nerve reinnervation, muscle atrophy was alleviated and the neuromuscular junctions were improved. On the whole, S-ketamine treatment achieved the structural enhancement of the motor neural circuit after BPRA in mice. As a result, mice in the S-ketamine group behaved better in the grooming test, CatWalk, and grip strength test.

The importance of ERK/CREB signaling via synaptic NMDARs is well-known for neuron survival and signal transduction under physical conditions, but it was silenced under pathological conditions due to the activation of extrasynaptic NMDARs at the same time (Parsons and Raymond, 2014; Hardingham et al., 2002). We demonstrated a reduction in ERK/CREB phosphorylation after BPRA in the control group and a reversal of this effect in the S-ketamine group.

Previous studies found that BDNF is a key element in neural repair after injury. In a rat facial nerve axotomy model, the transcription and translation levels of BDNF and its receptor TrkB significantly increased immediately after injury. BDNF expression levels increased several-fold within 24 h and reached the peak within 5–7 days after injury (Kobayashi et al., 2006). In addition, in a unilateral ventral funiculus cut rat model, the mesenchymal stem cells can increase the expression of BDNF at the injury site, thereby enhancing neuronal survival and promoting axonal regeneration, along with alleviating the excessive activation of astrocytes, with more pronounced effects on long-term neurological recovery (Spejo et al., 2018). In addition, the injection of the TNFα/TNF receptor-binding inhibitor R7050 can enhance ganglion cell survival by increasing BDNF levels, but its effect is still inferior to that of BDNF injected alone in the optic nerve injury model (Lucas-Ruiz et al., 2019). Finally, BDNF secretion could influence the effect of the therapeutic electrical stimulation for sciatic nerve transection and repair in rats (Kennedy et al., 2025). It is evident that BDNF is a key factor in neuronal survival, axonal regeneration, and the recovery of neurological function, playing a crucial role in the repair of the nervous system after injury. To verify that BDNF is a key factor in S-ketamine treatment of BPRA injury, we utilized ANA12, the antagonist of the BDNF receptor TrkB. Our results show that ANA12 counteracted the motor function recovery of S-ketamine treatment alone. The above evidence proves that BDNF plays an irreplaceable role in S-ketamine’s effect on BPRA injury.

Ketamine is a racemic mixture composed of (S)-ketamine (S-ketamine) and (R)-ketamine, while S-ketamine is the right-handed enantiomer. Ketamine is regarded as an immunomodulator due to its anti-inflammatory effects. A single subanesthetic dose of ketamine injection could effectively reduce the inflammatory factor level after surgery (Beilin et al., 2007). Ketamine can inhibit the production of pro-inflammatory cytokines induced by immune responses, such as nuclear factor kappa B (NF-κB), tumor necrosis factor alpha (TNFα), interleukin-6 (IL6), C-reactive protein, and nitric oxide synthase (iNOS) (Beilin et al., 2007; Larsen et al., 1998; Kawasaki et al., 1999; Kawasaki et al., 2001; Lankveld et al., 2005; Yang et al., 2005). In addition, ketamine was reported as a neuroprotector. NMDA receptor antagonists, such as MK-801, CQNX, and ketamine, can inhibit the increase in nitric oxide-dependent cyclic guanosine monophosphate (c-GMP) levels in rat cortical neurons induced by glutamate or glutamate analogs (Gonzales et al., 1995). Both racemic ketamine and S-ketamine can enhance the survival rates of neurons exposed to glutamate and those subjected to axonal transection injury, with S-ketamine also promoting axonal regeneration (Himmelseher et al., 1996). Further studies have shown that S-ketamine offers greater protective effects against glutamate exposure than racemic ketamine, primarily due to its ability to alleviate the dramatic decline in growth-associated protein-43 (GAP-43) and synaptosomal-associated protein 25 (SNAP-25) levels (Himmelseher et al., 2000). S-ketamine can increase hemoglobin oxygen saturation in cortical neurons, thereby reducing neuronal death after ischemic stroke in a rat model (Proescholdt et al., 2001). Additionally, S-ketamine exerts neuroprotective effects in rats with spinal cord injury (SCI) by lowering the level of subacute lipid peroxidation (LPO) (Kose et al., 2012). In our experiment, we have confirmed the protective effect of S-ketamine on spinal cord neurons after BPRA, mainly via the increase in the BDNF concentration.

S-ketamine, an anesthetic and psychoactive drug, is frequently associated with adverse effects such as headache, dissociation, and nausea in clinical use (Singh et al., 2016). However, there are no criteria to assess such symptoms in mice. Therefore, clinical human research studies focusing on the adverse effects of subanesthetic use of S-ketamine are needed in the future. Moreover, we have not paid attention to whether the mice exhibited head-nodding behaviors similar to those observed after ketamine abuse. Finally, the effective and safe range of a subanesthetic dose of S-ketamine on neural protection has not yet been investigated. Therefore, further considerate work is required in the future.

## Conclusion

S-ketamine increases the BDNF concentration by reversing ERK/CREB phosphorylation; therefore, it promotes motor neural circuit repair and facilitates motor function recovery.

## Data Availability

The raw data supporting the conclusion of this article will be made available by the authors, without undue reservation.

## References

[B1] Aleksandrova L. R. Phillips A. G. (2021). Neuroplasticity as a convergent mechanism of ketamine and classical psychedelics. Trends Pharmacol. Sci. 42 (11), 929–942. 10.1016/j.tips.2021.08.003 34565579

[B2] Beilin B. Rusabrov Y. Shapira Y. Roytblat L. Greemberg L. Yardeni I. Z. (2007). Low-dose ketamine affects immune responses in humans during the early postoperative period. Br. J. Anaesth. 99 (4), 522–527. 10.1093/bja/aem218 17681970

[B3] Benninger Y. Colognato H. Thurnherr T. Franklin R. J. M. Leone D. P. Atanasoski S. (2006). Beta1-integrin signaling mediates premyelinating oligodendrocyte survival but is not required for CNS myelination and remyelination. J. Neurosci. 26 (29), 7665–7673. 10.1523/JNEUROSCI.0444-06.2006 16855094 PMC6674273

[B4] Chen S. Hou Y. Zhao Z. Luo Y. Lv S. Wang Q. (2019). Neuregulin-1 accelerates functional motor recovery by improving motoneuron survival after brachial plexus root avulsion in mice. Neuroscience 404, 510–518. 10.1016/j.neuroscience.2019.01.054 30731156

[B5] Daly E. J. Trivedi M. H. Janik A. Li H. Zhang Y. Li X. (2019). Efficacy of esketamine nasal spray plus oral antidepressant treatment for relapse prevention in patients with treatment-resistant depression: a randomized clinical trial. JAMA Psychiatry 76 (9), 893–903. 10.1001/jamapsychiatry.2019.1189 31166571 PMC6551577

[B6] Duval T. Le Vy S. Stikov N. Campbell J. Mezer A. Witzel T. (2017). g-Ratio weighted imaging of the human spinal cord *in vivo* . NeuroImage 145, 11–23. 10.1016/j.neuroimage.2016.09.018 27664830 PMC5179300

[B7] Eggers R. de Winter F. Tannemaat M. R. Malessy M. J. A. Verhaagen J. (2020). GDNF gene therapy to repair the injured peripheral nerve. Front. Bioeng. Biotechnol. 8, 583184. 10.3389/fbioe.2020.583184 33251197 PMC7673415

[B8] Faglioni W. Siqueira M. G. Martins R. S. Heise C. O. Foroni L. (2013). The epidemiology of adult traumatic brachial plexus lesions in a large metropolis. Acta Neurochir. 156 (5), 1025–1028. 10.1007/s00701-013-1948-x 24318512

[B9] Fang X.-Y. Zhang W. M. Zhang C. F. Wong W. M. Li W. Wu W. (2016). Lithium accelerates functional motor recovery by improving remyelination of regenerating axons following ventral root avulsion and reimplantation. Neuroscience 329, 213–225. 10.1016/j.neuroscience.2016.05.010 27185485

[B10] Gonzales J. M. Loeb A. L. Reichard P. S. Irvine S. (1995). Ketamine inhibits Glutamate-N-Methyl-D-Aspartate-and quisqualate-stimulated cGMP production in cultured cerebral neurons. Anesthesiology 82 (1), 205–213. 10.1097/00000542-199501000-00025 7832303

[B11] Guo W.-L. Qu W. R. Zeng L. N. Qi Z. P. Huang C. Zhu Z. (2019). l-Theanine and NEP1-40 promote nerve regeneration and functional recovery after brachial plexus root avulsion. Biochem. Biophysical Res. Commun. 508 (4), 1126–1132. 10.1016/j.bbrc.2018.11.124 30553451

[B12] Hardingham G. E. Bading H. (2010). Synaptic versus extrasynaptic NMDA receptor signalling: implications for neurodegenerative disorders. Nat. Rev. Neurosci. 11 (10), 682–696. 10.1038/nrn2911 20842175 PMC2948541

[B13] Hardingham G. E. Chawla S. Cruzalegui F. H. Bading H. (1999). Control of recruitment and transcription-activating function of CBP determines gene regulation by NMDA receptors and L-Type calcium channels. Neuron 22 (4), 789–798. 10.1016/s0896-6273(00)80737-0 10230798

[B14] Hardingham G. E. Fukunaga Y. Bading H. (2002). Extrasynaptic NMDARs oppose synaptic NMDARs by triggering CREB shut-off and cell death pathways. Nat. Neurosci. 5 (5), 405–414. 10.1038/nn835 11953750

[B15] Himmelseher S. Pfenninger E. Georgieff M. (1996). The effects of ketamine-isomers on neuronal injury and regeneration in rat hippocampal neurons. Anesth. and Analgesia 83 (3), 505–512. 10.1097/00000539-199609000-00011 8780271

[B16] Himmelseher S. Pfenninger E. Kochs E. Auchter M. (2000). S(+)-Ketamine Up-Regulates neuronal regeneration associated proteins following glutamate injury in cultured rat hippocampal neurons. J. Neurosurg. Anesthesiol. 12 (2), 84–94. 10.1097/00008506-200004000-00003 10774601

[B17] Hui Y. Yan Z. Yang H. Xu X. Yuan W. E. Qian Y. (2022). Graphene family nanomaterials for stem cell neurogenic differentiation and peripheral nerve regeneration. ACS Appl. Bio Mater. 5 (10), 4741–4759. 10.1021/acsabm.2c00663 36102324

[B18] Ivanov A. Pellegrino C. Rama S. Dumalska I. Salyha Y. Ben-Ari Y. (2006). Opposing role of synaptic and extrasynaptic NMDA receptors in regulation of the extracellular signal‐regulated kinases (ERK) activity in cultured rat hippocampal neurons. J. Physiology 572 (3), 789–798. 10.1113/jphysiol.2006.105510 PMC1779993 16513670

[B19] Kaijomaa M. Gissler M. Äyräs O. Sten A. Grahn P. (2022). Impact of simulation training on the management of shoulder dystocia and incidence of permanent brachial plexus birth injury: an observational study. BJOG An Int. J. Obstetrics and Gynaecol. 130 (1), 70–77. 10.1111/1471-0528.17278 PMC10087175 36052568

[B20] Kavalali E. T. Monteggia L. M. (2012). Synaptic mechanisms underlying rapid antidepressant action of ketamine. Am. J. Psychiatry 169 (11), 1150–1156. 10.1176/appi.ajp.2012.12040531 23534055

[B21] Kawasaki C. Kawasaki T. Ogata M. Nandate K. Shigematsu A. (2001). Ketamine isomers suppress superantigen-induced proinflammatory cytokine production in human whole blood. Can. J. Anesthesia/Journal Can. d'anesthésie 48 (8), 819–823. 10.1007/BF03016701 11546726

[B22] Kawasaki T. Ogata M. Kawasaki C. Ogata J. Inoue Y. Shigematsu A. (1999). Ketamine suppresses proinflammatory cytokine production in human whole blood *in vitro* . Anesth. and Analgesia 89 (3), 665–669. 10.1097/00000539-199909000-00024 10475301

[B23] Kennedy V. Long M. D. Walters J. Adewuyi A. A. Franz C. K. (2025). Applications of advances in therapeutic electrical stimulation techniques and technologies in precision peripheral nerve repair: a narrative review. Adv. Technol. Neurosci. 2 (2), 97–101. 10.4103/atn.atn-d-24-00023 40620882 PMC12226930

[B24] Kobayashi N. R. Bedard A. M. Hincke M. T. Tetzlaff W. (2006). Increased expression of BDNF and trkB mRNA in rat facial motoneurons after axotomy. Eur. J. Neurosci. 8 (5), 1018–1029. 10.1111/j.1460-9568.1996.tb01588.x 8743749

[B25] Kose E. A. Bakar B. Ayva S. K. Kilinc K. Apan A. (2012). Neuroprotective effects of racemic ketamine and (S)-ketamine on spinal cord injury in rat. Injury 43 (7), 1124–1130. 10.1016/j.injury.2012.02.022 22436574

[B26] Lai T. W. Zhang S. Wang Y. T. (2014). Excitotoxicity and stroke: identifying novel targets for neuroprotection. Prog. Neurobiol. 115, 157–188. 10.1016/j.pneurobio.2013.11.006 24361499

[B27] Lankveld D. P. K. Bull S. Van Dijk P. Fink-Gremmels J. Hellebrekers L. J. (2005). Ketamine inhibits LPS-induced tumour necrosis factor-alpha and interleukin-6 in an equine macrophage cell line. Veterinary Res. 36 (2), 257–262. 10.1051/vetres:2004061 15720977

[B28] Larsen B. Hoff G. Wilhelm W. Buchinger H. Wanner G. A. Bauer M. (1998). Effect of intravenous anesthetics on spontaneous and endotoxin-stimulated cytokine response in cultured human whole blood. Anesthesiology 89 (5), 1218–1227. 10.1097/00000542-199811000-00023 9822011

[B29] Lucas-Ruiz F. Galindo-Romero C. Salinas-Navarro M. González-Riquelme M. J. Vidal-Sanz M. Agudo Barriuso M. (2019). Systemic and intravitreal antagonism of the TNFR1 signaling pathway delays axotomy-induced retinal ganglion cell loss. Front. Neurosci. 13, 1096. 10.3389/fnins.2019.01096 31680831 PMC6803525

[B30] Lundborg G. Rosén B. (2007). Hand function after nerve repair. Acta Physiol. 189 (2), 207–217. 10.1111/j.1748-1716.2006.01653.x 17250571

[B31] McIntyre R. S. Rosenblat J. D. Nemeroff C. B. Sanacora G. Murrough J. W. Berk M. (2021). Synthesizing the evidence for ketamine and esketamine in treatment-resistant depression: an international expert opinion on the available evidence and implementation. Am. J. Psychiatry 178 (5), 383–399. 10.1176/appi.ajp.2020.20081251 33726522 PMC9635017

[B32] Parsons M. P. Raymond L. A. (2014). Extrasynaptic NMDA receptor involvement in central nervous system disorders. Neuron 82 (2), 279–293. 10.1016/j.neuron.2014.03.030 24742457

[B33] Pisarchik A. N. Gerasimova S. A. Lebedeva A. V. Levanova T. A. Malkov A. E. Mikhaylov A. N. (2024). Advanced neuromorphic engineering approaches for restoring neural activity after brain injury: innovations in regenerative medicine. Regen. Med. Rep. 1 (2), 195–210. 10.4103/regenmed.regenmed-d-24-00012

[B34] Proescholdt M. Heimann A. Kempski O. (2001). Neuroprotection of S(+) ketamine isomer in global forebrain ischemia. Brain Res. 904 (2), 245–251. 10.1016/s0006-8993(01)02465-9 11406122

[B35] Reif A. Bitter I. Buyze J. Cebulla K. Frey R. Fu D. J. (2023). Esketamine nasal spray versus quetiapine for treatment-resistant depression. N. Engl. J. Med. 389 (14), 1298–1309. 10.1056/NEJMoa2304145 37792613

[B36] Singh J. B. Fedgchin M. Daly E. Xi L. Melman C. De Bruecker G. (2016). Intravenous esketamine in adult treatment-resistant depression: a double-blind, double-randomization, placebo-controlled study. Biol. Psychiatry 80 (6), 424–431. 10.1016/j.biopsych.2015.10.018 26707087

[B37] Spejo A. B. Chiarotto G. B. Ferreira A. D. F. Gomes D. A. Ferreira R. S. Jr Barraviera B. (2018). Neuroprotection and immunomodulation following intraspinal axotomy of motoneurons by treatment with adult mesenchymal stem cells. J. Neuroinflammation 15 (1), 230. 10.1186/s12974-018-1268-4 30107848 PMC6092804

[B38] Tang Y. Wang J. Wan S. Luo L. Qiu Y. Jiang S. (2019). Epigallocatechin gallate enhances the motor neuron survival and functional recovery after brachial plexus root avulsion by regulating FIG4. Folia Neuropathol. 57 (4), 340–347. 10.5114/fn.2019.90819 32337947

[B39] Wang H. Peng R.-Y. (2016). Basic roles of key molecules connected with NMDAR signaling pathway on regulating learning and memory and synaptic plasticity. Mil. Med. Res. 3 (1), 26. 10.1186/s40779-016-0095-0 27583167 PMC5006437

[B40] Wu G.-Y. Deisseroth K. Tsien R. W. (2001). Activity-dependent CREB phosphorylation: convergence of a fast, sensitive calmodulin kinase pathway and a slow, less sensitive mitogen-activated protein kinase pathway. Proc. Natl. Acad. Sci. 98 (5), 2808–2813. 10.1073/pnas.051634198 11226322 PMC30221

[B41] Yang J. Li W. Duan M. Zhou Z. Lin N. Wang Z. (2005). Large dose ketamine inhibits lipopolysaccharide-induced acute lung injury in rats. Inflamm. Res. 54 (3), 133–137. 10.1007/s00011-004-1334-5 15883747

[B42] Yao X. Kong L. Qiao Y. Brand D. Li J. Yan Z. (2024). Schwann cell-secreted frizzled-related protein 1 dictates neuroinflammation and peripheral nerve degeneration after neurotrauma. Cell Rep. Med. 5 (11), 101791. 10.1016/j.xcrm.2024.101791 39426375 PMC11604536

[B43] Yao X. Yan Z. Wang X. Jiang H. Qian Y. Fan C. (2021). The influence of reduced graphene oxide on stem cells: a perspective in peripheral nerve regeneration. Regen. Biomater. 8 (4), rbab032. 10.1093/rb/rbab032 34188955 PMC8226110

[B44] Yu A.-X. Wang Z. Yi X.-Z. (2023). Regenerative peripheral nerve interface prevents neuroma formation after peripheral nerve transection. Neural Regen. Res. 18 (4), 814–818. 10.4103/1673-5374.353498 36204848 PMC9700115

[B45] Yu L. Liu M. Li F. Wang Q. Wang M. So K. F. (2023). Celsr2 knockout alleviates inhibitory synaptic stripping and benefits motoneuron survival and axon regeneration after branchial plexus avulsion. Mol. Neurobiol. 60 (4), 1884–1900. 10.1007/s12035-022-03198-3 36593433 PMC9984348

[B46] Zanos P. Gould T. D. (2018). Mechanisms of ketamine action as an antidepressant. Mol. Psychiatry 23 (4), 801–811. 10.1038/mp.2017.255 29532791 PMC5999402

